# Scientific writing capacity building with early career researchers during study implementation: The Enterics for Global Health seven-country experience

**DOI:** 10.1371/journal.pgph.0006589

**Published:** 2026-06-12

**Authors:** Sonia I. Rao, Beth A. Tippett Barr, Erika Feutz, Kirsten Vannice, Patricia B. Pavlinac

**Affiliations:** 1 Department of Global Health, University of Washington, Seattle, Washington, United States of America; 2 Nyanja Health Research Institute, Salima, Malawi; 3 Kamuzu University of Health Sciences, Blantyre, Malawi; 4 The Gates Foundation, Seattle, Washington, United States of America; PLOS: Public Library of Science, UNITED STATES OF AMERICA

The advancement of early-career investigators (ECIs) pursuing research careers is highly dependent on their ability to secure grant funding and first-author publications in peer-reviewed journals [1]. A network of mentors and sponsors to guide these activities is critical to professional advancement [1–3]. During study implementation, primary study activities and analyses often take precedent over mentorship of ECIs in scientific writing, leaving ECIs to engage with the data only after the study and funding have ended and senior investigators and analysts have moved on. In global health research, where study implementation often takes place in low- and middle-income countries (LMICs), ECIs from LMICs are particularly underrepresented in published literature, especially in first and last author positions [4–7].

The Enterics for Global Health (EFGH) *Shigella* surveillance study is a research consortium establishing the incidence and consequences of *Shigella* diarrhea in Bangladesh, Kenya, Malawi, Mali, Pakistan, Peru, and The Gambia [8]. The consortium includes investigators from academic and research institutions in implementing countries and partner institutions in the United States and United Kingdom (Fig 1). During the development phase of EFGH, ECIs from the seven implementing country institutions were surveyed to solicit input on capacity building and training priorities. Scientific writing and data analysis consistently ranked as the highest priorities. Mentorship and funded training opportunities were identified as key facilitators. Lack of protected time, writing and analytic skills were key barriers.

**Fig 1:**
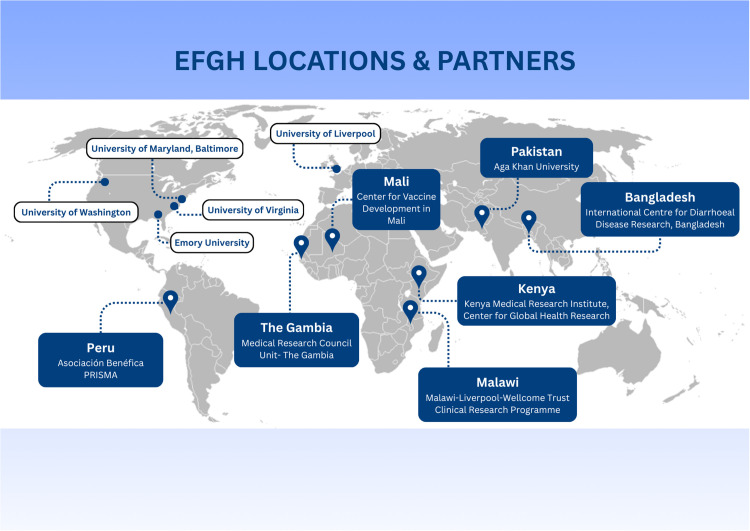
Enterics for Global Health (EFGH) Consortium Partners. Base image file: https://upload.wikimedia.org/wikipedia/commons/0/01/Blank-map-world-iceless-Greenland.png.

In response, we embedded a teaching and mentoring program to support EFGH ECIs to lead secondary analyses and first-author manuscripts using data collected in the primary study.

## Program structure

Eleven ECIs were selected for the manuscript writing certificate program (MWCP) through a competitive application process with efforts to balance gender and country site representation. The curriculum (Table 1) was delivered over 16 months in monthly online meetings supplemented with “office hours” hosted by program facilitators. Facilitators included an epidemiologist and research coordinator from the University of Washington as well as senior epidemiologist mentors from Nyanja Health institute. Participants were matched with an analysis topic of their choosing and a 3–4-person mentorship team. We solicited volunteer mentors from within the EFGH consortium to serve as primary, secondary, and data analyst mentors. Roles, responsibilities, and authorship expectations were set and agreed to via mentoring agreements (S1-S2 Appendices) at the beginning of the program. Data mentors provided a range of support, including giving input on the selection of variables and statistical tests, providing feedback on shell tables, coding and computing the analyses, or developing tables and figures. Most ECIs in the program opted for maximum data analysis support (S3 Appendix). Final deliverables of the program included a poster presentation delivered at an EFGH consortium in-person meeting, an oral presentation delivered to EFGH investigators, funders, and key stakeholders, and a first-authored manuscript published as part of this grouping of research papers.

**Table 1:** Manuscript Writing Certificate Program (MWCP) 16-month curriculum.

	Topics	Milestones
**1**	Program overview, participant goals and expectations High-level epidemiology concepts Formulating research questions	Identification of top 3 secondary analysis topics & formulating into testable research questions
**2**	Anatomy of research articles Literature review	Finalization of research topic and outlining of potential exposure, outcome & confounding variables
**3**	Anatomy of a research proposal part 1: specific aims page	Mentorship teams formed and mentorship agreements signed
**4**	Anatomy of a research proposal part 2: background/introduction section	Specific aims
**5**	Statistical Analysis Plans Part 1: variable definition table, basic statistical tests	Aims presentation to EFGH consortium
**6**	Statistical Analysis Plans Part 2: sample size/power calculations Anatomy of a research proposal part 3: methods section	Draft statistical analysis plans (SAP) due
**7**	Shell/dummy table development Data organization/presentation	Completed research proposal
**8**	Concept note submission and data request process	Data requests submitted to central data team & manuscript shell tables drafted
**9**	Data analysis: Importing and tabulating, univariate and multivariate correlates analyses	Practice in R using publicly available dataset
**10**	Anatomy of a manuscript: Introduction & methods	Introduction & methods section of manuscript
**11**	Anatomy of a manuscript: Results, tables and figures	Interim analysis (shell tables infilled)
**12**	Anatomy of a manuscript: Results, text	Results section of manuscript
**13**	Poster design	Results presentation to EFGH consortium
**14**	Anatomy of a manuscript: Discussion and abstract	Discussion outline
**15**	Designing and delivering effective presentations	Compiled manuscript draft
**16**	Final presentations	8-minute PowerPoint presentation

## Program outcomes

We measured participant progress using written assignments (S4-S10 Appendices), a mid-program survey, post-program survey, and group and individual feedback sessions.

All participants had learned English as a second or third language. None had previously first-authored a manuscript, and six had never co-authored a manuscript. All 11 participants successfully wrote and implemented a research proposal for secondary analysis of EFGH data, learned the basics of RStudio [9], developed posters, and drafted manuscripts for submission to a peer-reviewed journal. The program received extensive positive participant feedback:

*“It’s given me confidence and self-esteem, like how well you think you can do things in a more autonomous and independent way. And that’s a huge step in my career development. Before this program I didn’t know how to do that. I just watched the team do it and didn’t know how they did it. Now I can think of a topic, look for information on it, and develop a research protocol based on the gaps.” – MWCP participant*

Recommendations for improvement included more frequent and in-person sessions and ensuring participants’ supervisors allowed protected working time for the program. 80% of participants reported a desire for additional training in data analysis and interpretation.

## Lessons learned and future considerations

The MWCP feedback demonstrates the significant value of training and mentorship programs for LMIC-based ECIs and the strong desire for continued initiatives around scientific writing and analysis. While most published training interventions on manuscript writing average five days in length [1], we found that a longer-term, formalized approach was logistically and financially feasible while leveraging scientific expertise from our consortium and a strong facilitation team. While challenges arose, including coordinating around ongoing study activities and adapting training materials to participants with varying levels of research backgrounds, we received encouragement and reinforcement from the study funder. Clear, consistent, and frequent communication with mentors, especially by program facilitators, was necessary to ensure continued engagement over the length of the program. The amount of investment in terms of mentors’ time, from monthly meetings to reviewing drafts, is likely higher than in short-term workshop settings. For example, the availability of data analysts to not only support, but in most cases fully execute the statistical analysis plan, enabled more efficient progress and ability for the mentee to focus on scientific writing. The facilitators remained flexible to changing needs, including modifying the curriculum and timelines when certain topics required further attention. The cohort did not meet in-person until the end of the program, when a final 3-day writing workshop was held. Many participants cited this gathering as the most effective session of the program. If it had been feasible to organize more in-person sessions throughout the 16 months, or at a minimum to have started the program with an in-person meeting, we believe it would have further benefited participant engagement and learning outcomes.

## Conclusion

This program was feasible and successful at taking ECIs from the process of conceptualization through completion of a scientific manuscript. The intentional and proactive engagement of ECIs in a long-term, structured learning program which runs concurrently with the parent study can increase research capacity at sites, generate scientific outputs that raise the profile of the research, and fill global knowledge gaps.

## Supporting information

S1 Appendix EFGH Manuscript writing certificate program- mentorship team roles. (PDF)

S2 Appendix EFGH manuscript writing certificate program mentorship agreement. (PDF)

S3 Appendix EFGH MWCP levels of data support. (PDF)

S4 Appendix Assignment- formulating research questions. (DOCX)

S5 Appendix Assignment- literature review matrix. (DOCX)

S6 Appendix Assignment- research proposal. (DOCX)

S7 Appendix Assignment- abridged statistical analysis plan. (DOCX)

S8 Appendix Assignment- Table 2 correlates analysis. (DOCX)

S9 Appendix R code for Table 2 correlates analysis assignment. (R)

S10 Appendix Stata code for Table 2 correlates analysis assignment. (DO)
