# Supplementary material for: Scientific writing capacity building with early career researchers during study implementation: The Enterics for Global Health seven-country experience
Source: PLOS Glob Public Health. 2026 Jun 12;6(6):e0006589. doi: 10.1371/journal.pgph.0006589 (PMC13262805; doi:10.1371/journal.pgph.0006589)
Supplement: S1 Appendix — (PDF) [file pgph.0006589.s001.pdf]

EFGH Manuscript Writing Certificate Program- Mentorship Team Roles

	Responsibilities	Suggested authorship
**Mentee**	• Commit to the full 16-month program, which includes monthly meetings, didactic trainings, practice presentations, and interim deliverable requests, together requiring approximately 2 to 4 hours per week (8-16 hours per month).<br>• Develop a workplan and timeline for accommodating the time commitment of the program.<br>• Communicate early and often with MWCP facilitators if obstacles arise regarding assignments or meeting attendance.<br>• Schedule and facilitate check-ins with mentors to discuss progress. Mentees and mentors should agree upon the frequency and duration of check-ins (recommended at least monthly with primary mentor).<br>• Co-develop an authorship plan with mentorship team.	First author
**Primary mentor**	• Review course syllabus and mentee's workplan to ensure understanding of timeline.<br>• Attend meetings as agreed upon with mentee (recommended to meet at least once monthly)<br>• Read proposal draft and provide comments to the mentee in a timely fashion. Mentee and mentor should agree upon timelines for requesting and sending feedback/edits.<br>• Approve research proposal.<br>• Read manuscript drafts and provide comments to the mentee in a timely fashion. Mentee and mentor should agree upon timelines for requesting and sending feedback/edits.<br>• Assist the mentee with IRB application or exemption process, as well as any other human subjects requirements, as needed.<br>• Co-develop an authorship plan with mentee and other members of the mentorship team.	Last or co-last author

**Secondary mentor (optional)**	• Review course syllabus and mentee's workplan to ensure understanding of timeline.<br>• Read proposal and manuscript drafts and provide comments to the mentee and primary mentor in a timely fashion, consistent with agreed upon timeline.<br>• Attend meetings requested by the mentee or primary mentor.<br>• Co-develop an authorship plan with mentee and other members of the mentorship team.	Co-last author
**Data analyst (optional)**	• Review course syllabus and mentee's workplan to ensure understanding of timeline.<br>• Read and provide feedback on the proposal (specifically analysis plan section).<br>• Read and provide feedback on the statistical analysis plan.<br>• Work individually with the student on data analysis aspects of the project (e.g., coding support, conducting analysis).<br>• Support development and provide feedback on interim analyses, tables and figures, as needed<br>• Attend meetings requested by the mentee or primary mentor.<br>• Co-develop an authorship plan with mentee and other members of the mentorship team.	Second author
**MWCP facilitators (Patty Pavlinac, Sonia Rao)**	• Lead the process of guiding the mentee through project completion, including facilitating monthly cohort meetings and drop-in "office hours".<br>• Review and provide feedback on interim assignments.<br>• Facilitate adherence to mentorship agreement as needed and assist with mediation if conflict arises.	Acknowledged, middle or none depending on level of support
