# Supplementary material for: Scientific writing capacity building with early career researchers during study implementation: The Enterics for Global Health seven-country experience
Source: PLOS Glob Public Health. 2026 Jun 12;6(6):e0006589. doi: 10.1371/journal.pgph.0006589 (PMC13262805; doi:10.1371/journal.pgph.0006589)
Supplement: S2 Appendix — (PDF) [file pgph.0006589.s002.pdf]

EFGH Manuscript Writing Certificate Program (MWCP) - Mentorship Agreement

The EFGH Manuscript Writing Certificate Program is a 16-month structured training and mentorship program intended to support first-author manuscripts led by early-career researchers within the EFGH Consortium using already collected data from the EFGH study. Participants in the program will be expected to attend and actively participate in monthly remote meetings, didactic trainings, practice presentations and will be required to submit various interim deliverables (short proposal, statistical analysis plan, dummy tables, workplan and timeline, manuscript draft) with one-on-one support from senior mentors in the Consortium, data analysts, and the Nyanja Institute group, as well as through peer-to-peer feedback. By the end of the program, participants will ideally have a draft first-author manuscript ready for co-author review and submission to a scientific journal.

Aims

- To promote the use of existing EFGH data by early career low- and middle-income country (LMIC) researchers within the EFGH Consortium to first author manuscripts
- To provide tailored data analysis, proposal, and manuscript writing mentorship
- To build a strong professional network of early career EFGH researchers

---

Thank you for volunteering to be the **primary mentor or data analysis mentor** for a member of the EFGH MWCP cohort. This mentorship agreement must be signed by both mentee and mentors to ensure there is a mutual understanding of roles and responsibilities, authorship, and timelines. As a reminder, each mentorship team will consist of a primary mentor, a data analyst, and an optional secondary mentor.

Compared to advising, mentoring is “a distinct and deeper engagement that is based on a thorough personal understanding of one’s mentee and that individual’s personal career aspirations. Mentoring very frequently includes some advising yet transcends advising in its provision of individual-specific information and bilateral engagement and interactions that include the offering of advice based on a deep personal understanding of the mentee’s cadre of prior experiences, strengths and weaknesses, personal aspirations, values, and professional goals.”[1]

Together, mentees and mentors should review and discuss the expectations outlined on the next page, complete the requested sections, and sign and submit the agreement by **XX date.**

---

[1] Montgomery, B. L. (2017). Mapping a mentoring roadmap and developing a supportive network for strategic career advancement. *Sage Open*, *7*(2), 2158244017710288.

Mentee responsibilities:

- Commit to the full 16-month program, which includes monthly meetings, didactic trainings, practice presentations, and interim deliverable requests, together requiring approximately 2 to 4 hours per week (8-16 hours per month).
- Develop a workplan and timeline for accommodating the time commitment of the program.
- Communicate early and often with MWCP facilitators if obstacles arise regarding assignments or meeting attendance.
- Schedule and facilitate check-ins with primary mentor to discuss progress. Mentees and mentors should agree upon the frequency and duration of check-ins (recommended monthly).
- Co-develop an authorship plan with mentorship team.

Primary mentor responsibilities:

- Commit to actively supporting mentee for the full 16-month program, including check-ins to discuss progress. Mentees and mentors should agree upon the frequency and duration of check-ins (recommended monthly).
- Review course schedule and mentee's workplan to ensure understanding of course timeline.
- Read proposal draft and provide comments to the mentee in a timely fashion. Mentee and mentor should agree upon timelines for requesting and sending feedback/edits.
- Approve research proposal.
- Read manuscript drafts and provide comments to the student in a timely fashion. Mentee and mentor should agree upon timelines for requesting and sending feedback/edits.
- Assist the mentee with IRB application or exemption process, as well as any other human subjects requirements, as needed.
- Co-develop an authorship plan with mentee and other members of the mentorship team.

Data analysis mentor responsibilities:

- Review course schedule and mentee's workplan to ensure understanding of timeline.
- Read and provide feedback on the proposal (specifically analysis plan section).
- Read and provide feedback on the statistical analysis plan.
- Work individually with the student on data analysis aspects of the project (e.g., coding support, conducting analysis).
- Support development and provide feedback on interim analyses, tables and figures, as needed
- Attend meetings requested by the mentee or primary mentor.
- Co-develop an authorship plan with mentee and other members of the mentorship team.

Please discuss the following questions and note down the agreed upon plan:

1. How often will the mentee and primary mentor meet? Who will send calendar invites, meeting reminders, and agenda items? (our recommendation is that mentee takes on this task)

2. How often will the mentee and data analyst meet? Will they join meetings with the primary mentor or have separate meetings with the mentee?

3. What is the agreed upon turnaround time for mentors to provide feedback on a draft sent by the mentee for their review? (E.g. one week, two weeks, etc.)

Please complete the below information, sign and date. Mentee should return completed form with all signatures to MWCP facilitators.

MWCP mentee

**Name:** Click or tap here to enter text.
**Site:** Click or tap here to enter text.
**Primary contact method (choose one)***:* ☐ email: Click or tap here to enter text. ☐ phone/WhatsApp: Click or tap here to enter text. ☐ other (specify):Click or tap here to enter text.
**Alternate contact method (choose one)***:* ☐ email: Click or tap here to enter text. ☐ phone/WhatsApp: Click or tap here to enter text. ☐ other (specify):Click or tap here to enter text.
**Signature***:*Click or tap here to enter text.**Date***:*Click or tap here to enter text.

Primary Mentor

**Name:** Click or tap here to enter text.

**Site/Institution***:* Click or tap here to enter text.
**Primary contact method (choose one):** ☐ email: Click or tap here to enter text. ☐ phone/WhatsApp: Click or tap here to enter text. ☐ other (specify):Click or tap here to enter text.
**Alternate contact method (choose one)***:* ☐ email: Click or tap here to enter text. ☐ phone/WhatsApp: Click or tap here to enter text. ☐ other (specify):Click or tap here to enter text.
**Signature***:*Click or tap here to enter text. **Date***:*Click or tap here to enter text.

Data Analysis Mentor

**Name:** Click or tap here to enter text.
**Site/Institution***:* Click or tap here to enter text.
**Primary contact method (choose one):** ☐ email: Click or tap here to enter text. ☐ phone/WhatsApp: Click or tap here to enter text. ☐ other (specify):Click or tap here to enter text.
**Alternate contact method (choose one)***:* ☐ email: Click or tap here to enter text. ☐ phone/WhatsApp: Click or tap here to enter text. ☐ other (specify):Click or tap here to enter text.
**Signature***:*Click or tap here to enter text. **Date***:*Click or tap here to enter text.
