# Supplementary material for: Scientific writing capacity building with early career researchers during study implementation: The Enterics for Global Health seven-country experience
Source: PLOS Glob Public Health. 2026 Jun 12;6(6):e0006589. doi: 10.1371/journal.pgph.0006589 (PMC13262805; doi:10.1371/journal.pgph.0006589)
Supplement: S3 Appendix — (PDF) [file pgph.0006589.s003.pdf]

Level of Data Support	Minimal – mentor serves as resource for analytic questions and reviews materials	Moderate – mentor serves as resource for analytic questions, reviews materials, and provides analytic support as needed	Maximum – mentor serves as primary analyst
**Description of activities**	• Reviews variables chosen by mentee for analysis<br>• Reviews SAP completed by mentee<br>• Reviews shell tables drafted by mentee<br>• Provides code checks as needed<br>• Review interim analysis results, tables and figures, and provides feedback before mentee runs final analysis	• Assists mentee with thinking through necessary variables and statistical tests to address their research question<br>• Reviews SAP completed by mentee<br>• Reviews shell tables drafted by mentee<br>• Provides code checks and/or writes code for requested descriptive and/or inferential statistical analysis<br>• Assists mentee with completing tables and developing figures<br>• Review interim analysis results, tables, and figures and provides feedback before mentee runs final analysis	• Guides mentee through deciding necessary variables and statistical tests to address their research question<br>• Reviews SAP completed by mentee<br>• Reviews shell tables drafted by mentee<br>• Writes code for descriptive and/or inferential statistical analysis<br>• Runs interim analysis and completes tables/figures<br>• Reviews interim results with mentee and runs final analysis
