# Supplementary material for: Scientific writing capacity building with early career researchers during study implementation: The Enterics for Global Health seven-country experience
Source: PLOS Glob Public Health. 2026 Jun 12;6(6):e0006589. doi: 10.1371/journal.pgph.0006589 (PMC13262805; doi:10.1371/journal.pgph.0006589)
Supplement: S4 Appendix — (DOCX) [file pgph.0006589.s004.docx]

# SESSION: Formulating Research Questions

**Assignment 1**

**Summary of exercise:** You will choose your top three topic ideas from the list of secondary analyses *(to be adapted to specific parent study)* or your own ideas and draft research questions for each. This will help set you up for writing specific aims and hypotheses.

**Due: October 5, 2023**

**Step 1. Choose topics**:

- 1. *Each cohort member should identify 3 topics of interest from the list.*
  2. *Consider, what is the relevance of this research topic? Why would it be appropriate for your interests, experience and skills? And what would having the answer to this question allow you or others to do?*

| **Broad research topics of interest** List chosen topics here, with #1 being your most preferred |
| --- |
| **1.**  **2.**  **3.** |

| **Step 2: For each topic, define the:** | | | |  |  |
| --- | --- | --- | --- | --- | --- |
|  | *Population* | *Exposure (independent variable(s))* | *Outcome (dependent variable)* | *Possible Confounder(s)* | *Study design* |
| 1. |  |  |  |  |  |
| 2. |  |  |  |  |  |
| 3. |  |  |  |  |  |

| **Step 3. Specify your research questions:** Draft a research question to fit each of the topics you have selected. Make this very specific – include the exposure (independent variable(s)), outcome (dependent variable), and population you will study. |
| --- |
| **1.**  **2.**  **3.** |
| **Public health relevance of the research questions (2-3 sentences): *****This will form the initial ideas for the background section of your proposal. |
| **1.**  **2.**  **3.** |

**Step 4: Draw DAGs:** Fill in the boxes for a DAG for each of the 3 potential research questions using the independent, dependent, and possible confounders you brainstormed in step 2.

[Confounder]

[Outcome/Dependent variable]

[Exposure/Independent variable]

[Confounder]

[Outcome/Dependent variable]

[Exposure/Independent variable]

[Confounder]

[Outcome/Dependent variable]

[Exposure/Independent variable]
