# Supplementary material for: Scientific writing capacity building with early career researchers during study implementation: The Enterics for Global Health seven-country experience
Source: PLOS Glob Public Health. 2026 Jun 12;6(6):e0006589. doi: 10.1371/journal.pgph.0006589 (PMC13262805; doi:10.1371/journal.pgph.0006589)
Supplement: S5 Appendix — (DOCX) [file pgph.0006589.s005.docx]

**SESSION: Literature Reviews**

**Assignment 2**

**Summary of exercise:** Conduct a brief literature review of your topic by finding at least 5-10 primary research articles and populating the below table. This is just a starting point- you will continue to search the literature throughout your research and writing process. The matrix is a common tool researchers use to briefly summarize articles. This will help you complete the Background section for your proposal and manuscript. It will also help you start thinking about measurement (how did previous researchers define the key variables (exposure, outcome, confounding) and your analysis plan.

Primary research reports are research articles reporting novel studies and their findings. They **are not** review papers, white papers, factsheets, or websites.

Be sure to save your articles with your chosen citation manager (e.g., Zotero, Endnote).

Part 1: Please describe your search strategy (add rows as needed). We recommend searching at least 2 databases for this initial assignment.

| **Database** | **Search string** |
| --- | --- |
|  |  |
|  |  |
|  |  |

Part 2: Populate the below table with your findings from at least 5-10 primary research articles.

| **Author and year of publication** | **Article title** | **Population studied** | **Study design** | **Key exposure(s)** | **Key outcome(s)** | **Confounder(s) evaluated** | **Key findings** | **Outstanding questions or future research discussed** | **Theme or subtheme (optional)** |
| --- | --- | --- | --- | --- | --- | --- | --- | --- | --- |
|  |  |  |  |  |  |  |  |  |  |
|  |  |  |  |  |  |  |  |  |  |
|  |  |  |  |  |  |  |  |  |  |
|  |  |  |  |  |  |  |  |  |  |
|  |  |  |  |  |  |  |  |  |  |
|  |  |  |  |  |  |  |  |  |  |
|  |  |  |  |  |  |  |  |  |  |
|  |  |  |  |  |  |  |  |  |  |
|  |  |  |  |  |  |  |  |  |  |
|  |  |  |  |  |  |  |  |  |  |
