# Supplementary material for: Scientific writing capacity building with early career researchers during study implementation: The Enterics for Global Health seven-country experience
Source: PLOS Glob Public Health. 2026 Jun 12;6(6):e0006589. doi: 10.1371/journal.pgph.0006589 (PMC13262805; doi:10.1371/journal.pgph.0006589)
Supplement: S6 Appendix — (DOCX) [file pgph.0006589.s006.docx]

**Deliverable: Research Proposal**

**Due dates:**

- Specific Aims page due December 3, 2023
- Revised/updated aims page due January 5, 2024
- Background and significance due January 14, 2024
- Methods due February 25, 2024
- Full proposal completed, reviewed, and approved by mentors by March 21, 2024

**Formatting guidelines:**

- Single- spaced, 11-point Arial font (normal spacing), 0.5” margins all around, and fully justified (straight line in right and left-hand columns).
- Full name should appear as a header on the top left-hand side of the document and date on the top right-hand side
- All references must be properly cited, in-line with the text, using the ICMJE/Vancouver-style referencing.
- Submitted as a Microsoft Word document via Moodle.

**Structure of the proposal:**

The proposal should not exceed 4 pages single spaced, not including the Proposal Cover Sheet or references. The following format should be used:

- **Proposal Cover Sheet**: Indicate proposed title, MWCP participant name, primary mentor name, secondary mentor name, data analysis mentor name, and date. Signature of the primary mentor will be required to indicate approval of the proposal.
- **Specific Aims (0.5 - 1 page)**: This section should start by summarizing the burden of the health issue and stating the public health significance of the topic. Address what is known about your topic such as the causes of the problem and existing interventions to address it and key gaps in evidence that the proposed analysis will help fill and indicate the importance of the specific information being sought through the analysis. Clearly state your proposed study and population in 1-2 sentences, and then list your specific research questions and hypotheses. Finish with 1-3 sentences on what can be learned from this research.
- **Background and Significance (0.5 - 1 page**): This should NOT be an extensive literature review description (although an extensive review of the literature should be conducted prior to writing this concise background section. Rather this section should demonstrate a solid understanding of the issue or topic and be concisely written. Describe the disease (outcome of interest, its burden and consequences, as well as key populations impacted by this disease) as well as the risk factors and key gaps in the literature, setting the stage for why your research question is important. Describe the scientific context for the study, briefly summarizing the existing body of knowledge and/or the context and issues to be addressed in the practice setting. It may be helpful to include a table summarizing key findings from previous studies (see Table 1 in example proposal).
- **Methods (1-2 pages)**:
  - **Parent study**: 1-2 sentence description of the EFGH study.
  - **Study design of nested study:** 1 sentence description of your study design.
  - **Study setting:** 1-2 sentence description of where data were collected from.
  - **Study participants:** 2-3 sentence description of participant inclusion and exclusion criteria (for your specific analysis, not for the parent study).
  - **Data collection:** 6-8 sentence description of procedures that study personnel took to collect data (e.g. participant identification, screening, enrollment, data collection instruments, etc.)
  - **Statistical analysis:** 6-8 sentence description of your data analysis plan. What variables will be included in your analysis and what descriptive and analytic statistics will you present? Which statistical model(s) will you use? What statistical software package will you use to analyze data?
  - **Study power:** 3-4 sentence description of your power calculations and summary table of estimations
- **Limitations (1 paragraph)**: Describe any limitations that are beyond the control or extent of the study (which have already been determined), that may affect results of the research or influence the interpretation of the study results.
- **Ethical considerations:** 1-2 sentences outlining the status of required ethical approvals.
- **References**: Provide citations to all literature references used in the proposal. Proposal must have appropriate and consistent in-text citing using ICMJE/Vancouver style.
