# Supplementary material for: Scientific writing capacity building with early career researchers during study implementation: The Enterics for Global Health seven-country experience
Source: PLOS Glob Public Health. 2026 Jun 12;6(6):e0006589. doi: 10.1371/journal.pgph.0006589 (PMC13262805; doi:10.1371/journal.pgph.0006589)
Supplement: S7 Appendix — (DOCX) [file pgph.0006589.s007.docx]

**Enterics for Global Health (EFGH) Manuscript Writing Cohort Program**

Abridged Statistical Analysis Plan Template

Version XX

## Background and Rationale

XXXX

## Aims

- XXXX
- XXXX

**Study Population**

| Study Population | XXX |
| --- | --- |
| Inclusion Criteria |  |
| Exclusion Criteria |  |

In a flow diagram, report the reasons individuals ended up in the analytic study population. Specifically report the number of individuals (indicated by x’s at this stage) and reasons for falling out who:

1. Were screened in parent study
2. Were enrolled in parent study
3. Were included in the analysis

**Data Analysis**

## Variable Definition Table

| Variable Description | Definition | Assessed (at enrollment, 4-week follow-up, 3-month follow-up, etc.) | Type (continuous, categorical, etc.) | Values (if categorical) OR realistic range (if continuous) |
| --- | --- | --- | --- | --- |
|  |  |  |  |  |
|  |  |  |  |  |
|  |  |  |  |  |
|  |  |  |  |  |
|  |  |  |  |  |

## Independent/exposure variable(s):

## XXX

Dependent/outcome variable(s):

- XXX

Potential confounding variable(s):

- XXX

Potential effect modifying variable(s):

- XXX

## Analysis Methods (include plans for sensitivity analyses, if applicable)

**Aim 1.** *Repeat the aim XXX*

*Statistical Analysis:* XXX

*Power/Sample Size (NOTE: TO BE COMPLETED AFTER FEBRUARY COHORT MEETING, CAN BE LEFT BLANK IN FIRST DRAFT):* XXX

**Aim 2 (if applicable).** *Repeat the aim XXX*

*Statistical Analysis:* XXX

*Power/Sample Size (NOTE: TO BE COMPLETED AFTER FEBRUARY COHORT MEETING, CAN BE LEFT BLANK IN FIRST DRAFT): XXX*

**Level of Statistical Significance**

All statistical tests will be two-sided using a 5% significance level (alpha of 0.05).

References
