# Supplementary material for: Scientific writing capacity building with early career researchers during study implementation: The Enterics for Global Health seven-country experience
Source: PLOS Glob Public Health. 2026 Jun 12;6(6):e0006589. doi: 10.1371/journal.pgph.0006589 (PMC13262805; doi:10.1371/journal.pgph.0006589)
Supplement: S8 Appendix — (DOCX) [file pgph.0006589.s008.docx]

| Characteristic | Acutely malnourished  (N =X)  n (%) or median (IQR) | Not acutely malnourished  (N = X)  n (%) or median (IQR) | Unadjusted Prevalence Ratio (95% CI) | P-value | Adjusted Prevalence Ratio (95% CI)* | P-value |
| --- | --- | --- | --- | --- | --- | --- |
| **Sociodemographic** |  |  |  |  |  |  |
| Age at enrollment, mo |  |  |  |  |  |  |
| 0 to 5 |  |  | ref | - | ref | - |
| 6 to 11 |  |  |  |  |  |  |
| 12 to 23 |  |  |  |  |  |  |
| 24 to 59 |  |  |  |  |  |  |
| Caregiver schooling no more than primary^†^ |  |  |  |  |  |  |
| **Admission History** |  |  |  |  |  |  |
| Discharge diagnoses^§^ |  |  |  |  |  |  |
| Anemia |  |  |  |  |  |  |
| Gastroenteritis/diarrhea |  |  |  |  |  |  |
| Lower Respiratory Tract Infection |  |  |  |  |  |  |
| Malaria |  |  |  |  |  |  |
| Malnutrition |  |  |  |  |  |  |
| Meningitis |  |  |  |  |  |  |
| Sepsis |  |  |  |  |  |  |
| Sickle Cell^‖^ |  |  |  |  |  |  |
| Tuberculosis |  |  |  |  |  |  |
| Duration of hospital admission, days |  |  |  |  |  |  |
| Received antibiotics in hospital |  |  |  |  |  |  |
| **Nutritional and Vaccine Status** |  |  |  |  |  |  |
| Stunted (HAZ < -2)^††^ |  |  |  |  |  |  |
| Received all age-appropriate vaccines^†¶¶^ |  |  |  |  |  |  |

**Table 2.** Correlates of acute malnutrition among Kenyan children recently discharged from hospital for non-traumatic conditions

* Adjusted for site and child age † Among those who had responses. § Diagnoses may not be mutually exclusive. Percentages are among the 1,330 children who had records available and diagnosis recorded. ‖ Includes sickle cell crisis and/or sickle cell disease as comorbidity as not always distinguished in medical record. †† HAZ= height-for-age z-score. Among those with plausible values. ¶¶ According to the Kenya Ministry of Health Vaccine schedule (allowing a 4-week window). Malaria vaccine and measles vaccine at 6 months for HIV positive children were not included in definition of “all age-appropriate vaccines” despite being recommended in Kenyan guidelines.
